# The physiochemical characteristics of Phragmites communis trin polysaccharides and *in vitro* anti-diabetic effects of their different fractions

**DOI:** 10.3389/fphar.2026.1727657

**Published:** 2026-04-01

**Authors:** Wei Gao, Kundian Che, Baogang Zhou, Ran Liu, Zhanjun Chen, Jialin Yang, Haoyuan Luo, Chengfei Huang, Jiayu Wang, Ke Feng, Sarengaowa, Wenzhong Hu

**Affiliations:** 1 College of Life Science, Zhuhai College of Science and Technology, Zhuhai, China; 2 College of Life Sciences, Jilin University, Changchun, China; 3 Faculty of Medicine, Macau University of Science and Technology, Macao, China

**Keywords:** diabetes treatment, insulin resistance model, polysaccharides, reed root, α-glucosidase inhibition

## Abstract

This study isolated and purified two polysaccharide fractions, PTP-1 and PTP-2, from *Phragmites communis* Trin root via cellulase-assisted ultrasonication and DEAE chromatography, with purities of 90.56% and 91.35%, respectively. Structural characterization revealed that PTP-2 possessed a higher uronic acid content (9.45%) compared to PTP-1 (3.0%), along with a hyperbranched architecture and superior thermal stability. High-performance gel permeation chromatography (HPGPC) indicated that PTP-1 and PTP-2 had weight-average molecular weights of 55.8 kDa and 59.6 kDa, respectively, reflecting distinct structural features. *In vitro* assays demonstrated that PTP-2 exhibited potent α-glucosidase inhibition (IC_50_ = 0.679 mg/mL) and significantly enhanced glucose uptake and glycogen synthesis in insulin-resistant HepG2 cells, attributable to its uronic acid-enriched structure. These findings highlight PTP-2 as a promising multi-target agent for diabetes management, underscoring the structure–activity relationship of plant polysaccharides.

## 1 Introduction

Polysaccharides are polymers composed of more than ten monosaccharides, linked by glycosidic bonds, and are widely distributed in nature (Ji et al., 2022). They are essential for the normal functioning of organisms, participating in various significant biological activities. Natural polysaccharides, which have low toxicity and minimal side effects, are primarily sourced from bacteria, plants, and fungi. China’s rich natural resources provide an advantageous environment for research and development of these polysaccharides (Ji et al., 2020a; 2020b). Advancements in technology have facilitated the integration of novel structural identification techniques and biological activity evaluation methodologies in polysaccharide research. This integration has significantly enhanced the depth of understanding concerning the structural and functional attributes of polysaccharides. Extensive research has established that these compounds possess multiple critical biological activities, including anti-tumor, blood sugar-lowering, antioxidative, and immunomodulatory effects (Min et al., 2024; Cui and Li, 2025). This paper focuses on the potential value and application prospects of natural polysaccharides with hypoglycemic activity in the nutraceutical and pharmaceutical industries.

In recent years, the prevalence of diabetes has been rising, with an increasing trend among younger individuals. Clinically, diabetes manifests as a glucose metabolism disorder, often accompanied by complications such as neuropathy, retinopathy, and renal disease, posing a severe public health threat (Qin et al., 2019; Dong et al., 2024). Additionally, diabetes imposes a significant burden on healthcare systems. Despite ongoing research, a definitive cure for diabetes for diabetes has yet to be discovered worldwide. Reducing postprandial blood sugar is a critical treatment approach. Conventional clinical treatments involve insulin injections and oral hypoglycemic drugs. These oral agents include biguanides, α-glucosidase inhibitors, thiazolidinediones, and sulfonylureas. However, some of these drugs can lead to adverse reactions; for instance, metformin may cause indigestion in certain patients, and sulfonylureas can increase the risk of weight gain and cardiovascular disease (Kaur et al., 2015). To mitigate these side effects, researchers are increasingly focusing on natural hypoglycemic substances, such as flavonoids, saponins, alkaloids, and polysaccharides.

For decades, polysaccharides have been applied in various fields, including pharmaceuticals, biomaterials, and food (Ji et al., 2023). There is growing recognition of the role of natural polysaccharides as structural components and energy sources in biological systems. Mounting evidence shows that natural polysaccharides are highly effective in lowering blood sugar, making them a focal point of recent research in the field of polysaccharide activity. Furthermore, studies have explored the mechanisms by which polysaccharides reduce blood sugar, as well as their positive effects on diabetes and its complications, highlighting the considerable potential of natural polysaccharides in glucose management. Mechanistically, polysaccharides exert hypoglycemic effects through multiple pathways, including inhibition of carbohydrate-hydrolyzing enzymes (α-glucosidase and α-amylase), enhancement of insulin sensitivity, promotion of glucose uptake in peripheral tissues, stimulation of hepatic glycogen synthesis, and alleviation of oxidative stress (Jiang et al., 2025).

Reed root, derived from Phragmites communis, is commonly uesd in traditional Chinese medicine. It is recognized for its properties in clearing heat, detoxifying the body, promoting diuresis, and lowering blood glucose levels. Historically, traditional Chinese medicine has documented the use of reed root for the management of conditions analogous to diabetes. Contemporary medical research has corroborated the presence of numerous bioactive constituents within reed root, including polysaccharides, alkaloids, and natural antioxidants (Ye et al., 2023). In this study, polysaccharides were extracted from reed rhizomes through a process involving cellulase-assisted ultrasonication. The inhibitory effects of these polysaccharides on the enzymatic activities of α-amylase and α-glucosidase were subsequently evaluated. Furthermore, an insulin resistance model using human liver cancer cells was established to assess the potential glucose-lowering effects of the reed rhizome polysaccharides. Therefore, this study not only elucidates the structure–activity relationship of reed root polysaccharides but also provides a scientific basis for developing PTP-2 as a natural, multi-target therapeutic candidate for diabetes, with potential applications in functional foods and nutraceuticals.

## 2 Materials and methods

### 2.1 Materials and reagents

Reed root (purchased from Zhuhai, Guangdong Province, China), monosaccharide and polysaccharide standards (Shanghai Yuan Ye Biotechnology Co., Ltd.), human liver cancer cells HepG2 (Qing Qi Biotechnology Development Co., Ltd., Cat. No. BFN60800692, Lot No. 20190326 AOF2), glucose detection kit (Shanghai Rong Sheng Biopharmaceutical Co., Ltd.), liver glycogen detection kit (Nanjing Jian Cheng Bioengineering Institute), SOD assay kit (Bi Yun Tian Biotechnology Co., Ltd.).

### 2.2 Extraction and purification of PTP

The optimal extraction conditions for Phragmites communis Trin polysaccharides (PTP) were determined through single-factor and response surface methodology experiments. Initially, a specified amount of Phragmites communis Trin powder was weighed, and sieved to a particle size of 0.25 mm. The extraction was then performed in an ultrasonic extractor (model XH-300A, Xiang Hu Technology, China) using an 18:1 solvent to solid ratio, a cellulase concentration of 0.4%, an ultrasonic power setting of 297W, and a duration of 24 min. Following extraction, the crude extract was centrifuged at 8000 rpm for 10 min to separate the supernatant which was then concentrated using rotary evaporation.

The concentrated extract underwent protein removal via the Savage metho (Luan et al., 2023), followed by dialysis and alcohol precipitation. The precipitate was redissolved and subsequently freeze-dried to yield the Phragmites communis Trin polysaccharides (PTP). For further purification, PTP was subjected to DEAE ion-exchange column chromatography. Fractions were collected based on their elution profiles, resulting in the isolation of two distinct polysaccharide components, designated PTP-1 and PTP-2. The polysaccharide detection was carried out using the phenol-sulfuric acid method.

### 2.3 Characterization of PTP

#### 2.3.1 Determination of molecular weight of PTP

The molecular weight of the components from polysaccharide purification was determined using the High-Performance Gel Permeation Chromatography (HPGPC) method. The experimental conditions were as follows: a TSK-gel G3000 PWXL chromatography column (7 μm, 7.8 × 300 mm) from TOSOH (Japan TSK), with the column temperature maintained at 40 °C. The system featured a differential refractive index detector, a flow rate of 0.5 mL/min, and a mobile phase consisting of 0.05 M sodium sulfate (He et al., 2016); The injection volume was 20 μL.

Standard Curve Construction: Dextran standards of varying molecular weights were prepared at a concentration of 4.0 mg/mL and analyzed using the HPGPC method described above. The retention time (T, in minutes) and the logarithm of the corresponding molecular weight (Log MW) were plotted on the x-axis and y-axis respectively to establish a standard curve. This resulted in a regression equation for determining molecular weight.

Log MW=2.3944T+23.289

#### 2.3.2 Analysis of monosaccharide composition of PTP

The monosaccharide composition was analyzed via pre-column derivatization with 1-phenyl-3-methyl-5-pyrazolone (PMP) followed by high-performance liquid chromatography (HPLC). Briefly, 10 mg of polysaccharide was hydrolyzed with 4 M trifluoroacetic acid at 110 °C for 4 h. The hydrolysate was derivatized with 0.5 M PMP in 0.3 M NaOH at 70 °C for 1 h, neutralized with HCl, and extracted with chloroform. The aqueous phase was filtered and analyzed using an Agilent Eclipse XDB-C18 column with gradient elution (acetonitrile–triethylamine) and detection at 254 nm.

#### 2.3.3 Ultraviolet spectrum

Prepare a solution of purified reed rhizome polysaccharide components at a concentration of 0.5 mg/mL. Scan the ultraviolet absorption spectrum of the polysaccharide solution within the wavelength range of 200–600 nm in ultraviolet-visible light (Han et al., 2023). The UV-Vis spectra were scanned from 200 to 600 nm because polysaccharides typically do not exhibit absorption above 600 nm, and this range is sufficient to detect potential protein (280 nm) and nucleic acid (260 nm) contaminants.

#### 2.3.4 Fourier transform infrared (FTIR) spectroscopy

The Fourier Transform Infrared (FT-IR) absorption spectra of PTP-1 and PTP-2 were determined using the potassium bromide (KBr) pellet method. A thoroughly dried 2.0 mg sample of PTP was mixed uniformly with 300 mg of KBr powder, and then finely ground using an agate mortar. Finally, the spectra within the range of 450–4000 cm-1 were scanned and obtained using an FT-IR instrument (Yue et al., 2023).

#### 2.3.5 Thermogravimetric analysis

Place 5.0 mg of each PTP-1 and PTP-2 sample into a platinum crucible and conduct real-time thermogravimetric analysis (TGA) under a nitrogen flow of 50 mL/min. Measure the mass loss rate of the polysaccharide samples within the temperature range of 25 °C–700 °C at a heating rate of 10 °C/min (Sanches et al., 2023).

#### 2.3.6 Atomic force microscopy (AFM)

The preparation of the polysaccharide sample to be tested was optimized according to the method of Yu et al. (2024). First, a stock solution of the purified polysaccharide component from reed rhizome was prepared at a concentration of 1 mg/mL, and magnetically stirred at 50 °C for 2 h to reduce the aggregation of the polysaccharides. Then, the stock solution was diluted to 10 μg/mL and subjected to magnetic stirring again. After filtration, a sample solution was obtained and an appropriate amount was taken and air-dried on a mica sheet. The conditions for Atomic Force Microscopy (AFM) determination were as follows: an un-dropped mica sheet was used as the scanning substrate, with an Si3N4 probe used to scan for micro-morphological characteristics of purified reed rhizome polysaccharide samples. Finally, AFM images were obtained.

#### 2.3.7 Scanning electron microscopic (SEM)

Take an appropriate amount of dried PTP-1 and PTP-2 samples and adhere them to the black double-sided adhesive tape on the sample stage. After gold sputtering, observe the morphological features of the samples in the scanning electron microscope chamber and capture images of representative regions (Wei et al., 2022).

### 2.4 Extracorporeal hypoglycemic activity

α-Amylase and α-glucosidase are pivotal digestive enzymes involved in carbohydrate metabolism in humans. α-Amylase catalyzes the hydrolysis of starch into smaller sugar units, such as maltose, whereas α-glucosidase primarily hydrolyzes non-reducing disaccharides (e.g., maltose, sucrose) into monosaccharides, such as glucose (Capetti et al., 2020). These monosaccharides are subsequently absorbed into the bloodstream, leading to elevated blood glucose levels.

In the management of hyperglycemia, inhibitors of α-amylase and α-glucosidase play a crucial role. These pharmacological agents impede the enzymatic activity of both enzymes, thus decelerating carbohydrate breakdown and sugar absorption rates (Abioye et al., 2022). This modulation helps mitigate sharp postprandial spikes in blood glucose levels, aiding in the glycemic control for individuals with diabetes.

#### 2.4.1 Inhibition of α-glucosidase by PTP

In this study, all experimental solutions were prepared using a 100 mM phosphate buffer solution at pH 6.9. Initially, an α-glucosidase solution was prepared to a final concentration of 0.5 U/mL. This enzyme solution was then mixed in equal volumes with a polysaccharide solution, which was prepared at varying concentration gradients. The resulting mixture was incubated at 37 °C for 10 min. Following incubation, an equal volume of p-nitrophenyl-α-D-glucopyranoside (pNPG) solution (also prepared in the same phosphate buffer to a final concentration of 5 mM) was added to initiate the enzymatic reaction. The reaction proceeded at 37 °C for an additional 20 min before being terminated by the addition of a 1 M sodium carbonate solution (Ren et al., 2024). Aliquots of the reaction mixture were then taken for absorbance measurement at 405 nm in order to calculate the inhibition rate of α-glucosidase using formula. Acarbose, used as a positive control at equivalent concentrations, and all samples were tested in triplicate to ensure experimental reliability and reproducibility.

$$\alpha -\text{Glucosidase inhibition rate }\left(\%\right)=\left[1-\left(\left(A-B\right)/C\right)\times 100\right]$$

Where A is the absorbance value for the mixture containing polysaccharide solution, enzyme solution and starch solution; B is the absorbance value for the mixture containing polysaccharide solution, buffer and starch; C is absorbance value for mixture containing buffer, enzyme and starch solutions.

#### 2.4.2 Inhibition of α-amylase by PTP

All reagents required for the experiment were prepared in a 20 mM phosphate buffer at pH 6.9. An α-amylase solution was formulated to a final concentration of 1.0 U/mL. To this solution, an equal volume of various test solutions at different concentrations was added and mixed thoroughly. The mixture was then incubated at 37 °C for 10 min. Subsequently, an equal volume of a 1% starch solution was added to the enzyme-sample mixture, mixed thoroughly to create reaction mixture 'a', and further incubated at 37 °C for an additional 10 min.To terminate the enzymatic reaction in mixture 'a', twice its volume of a 3,5-dinitrosalicylic acid color reagent was added, resulting in mixed solution 'b'. This solution 'b' was then heated in a boiling water bath for 5 minutes and subsequently diluted prior to measuring its absorbance at a wavelength of 540 nm (Faraone et al., 2019).

The inhibition rate of α-amylase activity by each sample group was calculated using formula. The experiment included a positive control group using acarbose at concentrations comparable to those of the test samples. All samples were tested in triplicate to ensure reliability and reproducibility of the results.

$$\alpha -\text{Glucosidase inhibition rate }\left(\%\right)=\left[1-\left(\left(A-B\right)/C\right)\times 100\right]$$

Where A is the absorbance value of the mixed solution containing polysaccharide solution, enzyme solution, and starch solution; B is the absorbance value of the mixed liquid containing polysaccharide solution, buffer, and starch; C is the absorbance value of mixed liquid containing buffer, enzyme solution, and starch.

#### 2.4.3 Cultivation and treatment of human hepatocellular carcinoma cells

Rapidly thaw cryopreserved cells by immersing them in a 37 °C water bath. Following thawing, centrifuge at appropriate g-forces to sediment the cells. Carefully decant the supernatant and resuspend the cell pellet in a high-glucose culture medium supplemented with 10% fetal bovine serum and 1% antibiotic-antimycotic solution. Ensure homogeneity in the cell suspension through gentle pipetting before seeding into a culture vessel containing 5 mL of the same medium formulation. Place the culture vessel in a CO_2_incubator set at 37 °C with a 5% CO_2_ atmosphere to promote optimal cell growth conditions.

After revival and 3–4 passages, HepG2 cells in the logarithmic growth phase should be evaluated for cell density. The cells should then be diluted to a concentration of 3 × 10^4^ cells/mL and seeded into a 96-well culture plate. Following a 24-h incubation to allow for cell recovery and adherence, the cells should be divided into various treatment groups. The experimental design includes a normal control group (comprising liver cancer cells in complete culture medium), a model control group (IR-HepG2 cells in complete culture medium), a positive control group (IR-HepG2 cells in metformin-supplemented medium), and an experimental group (IR-HepG2 cells treated with varying concentrations of polysaccharide-enriched medium) (Zhang et al., 2022).

#### 2.4.4 Cell viability assessment via CCK8 assay

After 3–4 passages following revival, assess whether the cells are in good growth condition. Select HepG2 cells that are in the logarithmic growth phase for further experimentation. Digest and collect the cells using trypsin-EDTA, then add 1 mL of complete culture medium to prepare a cell suspension. After counting the cells using a hemocytometer, dilute the cell suspension to a concentration of 3 × 10^4^ cells/mL. Add 100 μL of this suspension per well when seeding into a 96-well cell culture plate. Twenty-four hours after cultivation, evaluate the condition of the cells. Discard the culture medium from each well and replenish it with a fresh medium, each supplemented with different concentrations of ptp-1 and PTP-2 (1000 μg, 800 μg, 400 μg, 200 μg, 100 μg, and 50 μg/mL) (Gao, et al., 2015). The selection of the concentration range for polysaccharides is based on the preliminary results of cytotoxicity tests. Make sure to replicate each concentration in six wells. Twenty-four hours post-culturing, examine the condition of the cells under a microscope. In a light-protected environment, carefully remove the medium from each well of the 96-well plate and wash with PBS. Subsequently, add 110 μL of CCK-8 solution to each well. Allow incubation at 37 °C for 50 min, then remove and promptly measure the absorbance at 450 nm using a microplate spectrophotometer.

#### 2.4.5 The effect of PT polysaccharides on glucose consumption in hepatocellular carcinoma cells

The methodology for cell grouping and treatment is detailed in Section 4.4.3. Glucose consumption by cells in various treatment groups was quantified utilizing a glucose assay kit manufactured by Nanjing Jian Cheng, following the protocol specified in the accompanying instructions. The glucose consumption (GC) for each treatment group was determined using the formula outlined below:

$$\text{Glucose Uptake}=C_{1}v/C_{2}v\times 1000$$

Glucose uptake (mmol) C1: Initial glucose concentration in the culture medium (mol/L) C2: Glucose concentration in the culture solution after 24 h incubation (mol/L) V: Volume of the culture medium (L) The Effect of PT Polysaccharides on Glycogen Content in Hepatocellular Carcinoma Cells.

#### 2.4.6 The effect of PT polysaccharides on glycogen content in hepatocellular carcinoma cells

The method of cell grouping treatment is described in section 4.4.3. Cells are seeded into 12-well plates and subjected to various treatments. Post-treatment, cells from each group are harvested and washed 2-3 times with precooled phosphate-buffered saline (PBS). Subsequently, 225 μL of alkaline solution are added to each sample, followed by incubation in a boiling water bath for 20 min. After cooling the samples under running water, an additional 225 μL of double-distilled water are added to prepare the glycogen detection solution for subsequent analysis. The glycogen content within the cells is quantified using the Nanjing Jiancheng Glycogen Assay Kit according to the manufacturer’s instructions. The glycogen content is calculated using a specific formula provided in the protocol.

#### 2.4.7 The effect of PT polysaccharides on reactive oxygen species levels in hepatocellular carcinoma cells

The detailed protocols for cell grouping and processing are described in Section 2.4.3. Cells were seeded into 6-well plates and subjected to various treatments. After processing, cells from each group were collected and washed 2–3 times with pre-cooled phosphate-buffered saline (PBS). Subsequent steps were performed according to the instruction manual of the ROS detection kit: the DCFH-DA probe working solution was diluted to 10 μmol/L with basal medium at a ratio of 1:1000, and the diluted probe working solution was used to resuspend the cells. The cell suspension was incubated in a cell culture incubator for 20 min, with gentle inversion and mixing at 3–5-min intervals to ensure homogeneous contact between the probe and cells. Cells were then washed three times with serum-free medium, followed by resuspension in 1 mL of basal medium. Fluorescence intensity of cells from each group was finally measured using flow cytometry.

### 2.5 Statistical analysis

All experimental procedures were repeated three times. All data are presented as mean ± standard deviation (SD). Graphs were created using GraphPad Prism 10, and statistical analysis including one-way analysis of variance (ANOVA) and Duncan’s multiple range test was conducted using SPSS 27 software. P < 0.05 was considered as statistically significant.

## 3 Results

### 3.1 Analysis of extraction and purification contents

The crude polysaccharide content in the rhizomes of reeds was determined to be 325.24 ± 1.25 mg/g, using cellulase and ultrasonic extraction methods. Following purification via DEAE ion exchange chromatography, the polysaccharides extracted from reed rhizomes were designated as PTP-1 and PTP-2, with purities of 90.56% and 91.35% respectively. After undergoing freeze-drying, the samples were obtained as a brown powder.

### 3.2 Molecular weight determination results

PTP-1 exhibited a three-component profile: a dominant main peak (55.8 kDa, 78.5%), a secondary peak (1.4 kDa, 20.3%), and trace constituents (0.4 kDa, 1.2%), while PTP-2 contained only two components (main peak: 59.6 kDa, 77.3%; low-molecular-weight fraction: 1.7 kDa, 22.7%). The main chain molecular weights of the two polysaccharides differed by less than 7% (55.8 vs. 59.6 kDa), suggesting differentiated branching patterns between them. Notably, PTP-1 displayed a higher proportion of low-molecular-weight fragments (21.5% cumulative from secondary and trace peaks) compared to PTP-2 (22.7% from a single low-Mw peak), which may correspond to thermolabile side chains that are more susceptible to thermal degradation. PTP-1 exhibited a three-component profile: a dominant main peak (55.8 kDa, 78.5%), a secondary peak (1.4 kDa, 20.3%), and trace constituents (0.4 kDa, 1.2%), while PTP-2 contained only two components (main peak: 59.6 kDa, 77.3%; low-molecular-weight fraction: 1.7 kDa, 22.7%). The main chain molecular weights of the two polysaccharides differed by less than 7% (55.8 vs. 59.6 kDa), suggesting differentiated branching patterns between them. Notably, PTP-1 displayed a higher proportion of low-molecular-weight fragments (21.5% cumulative from secondary and trace peaks) compared to PTP-2 (22.7% from a single low-Mw peak), which may correspond to thermolabile side chains that are more susceptible to thermal degradation.

### 3.3 Monosaccharide composition analysis

**FIGURE 1:**
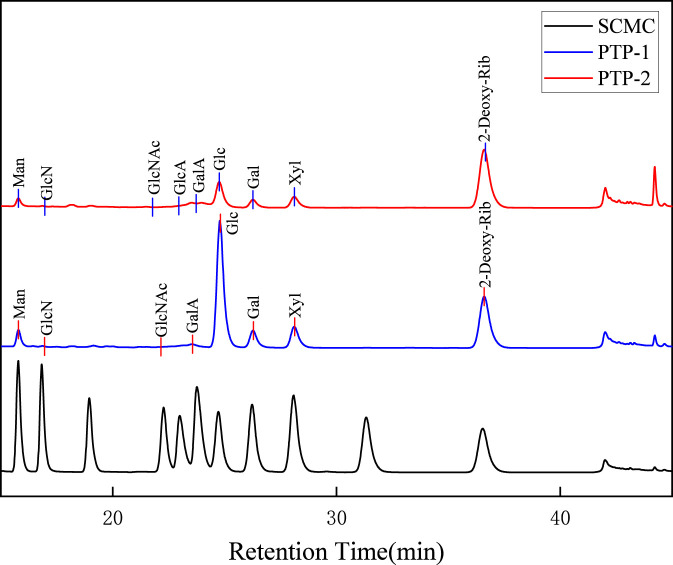
Monosaccharide composition profiles of PTP-1 and PTP-2.

The HPLC chromatograms depicting the monosaccharide composition of reed root polysaccharides PTP-1 and PTP-2 are illustrated in Figure 1. According to Figure 1, Tables 1, 2 the monosaccharide composition and relative molar ratios are detailed. PTP-1 consisted of mannose (5.48%), glucosamine (0.30%), N-acetylglucosamine (0.73%), galacturonic acid (3.00%), glucose (71.77%), galactose (9.03%), and xylose (9.70%). In contrast, PTP-2 contained mannose (8.27%), glucosamine (0.51%), N-acetylglucosamine (1.95%), glucuronic acid (0.45%), galacturonic acid (9.45%), glucose (50.87%), galactose (12.67%), and xylose (15.82%). While both polysaccharides share a similar monosaccharide profile; glucose remains the most abundant monomer in both cases. The total molar ratios for uronic acids in PTP-2 are significantly higher than those in PTP-1; this difference correlates with the observed intensities of C=O double bond vibrations in infrared spectroscopy analyses.

**TABLE 1:** The chemical component analysis of MEO.

Polysaccharide fraction	Peak no.	Retention time (min)	Molecular weight (kDa)	Relative peak area (%)
PTP-1	Peak1	11.923	55.8	78.5
	Peak2	15.724	1.4	20.3
	Peak3	17.19	0.4	1.2
PTP-2	Peak1	11.856	59.6	77.3
	Peak2	15.537	1.7	22.7

**TABLE 2:** Monosaccharide composition (%) and molar ratios of PTP-1 and PTP-2.

Polysaccharide fraction	Man	GlcN	Rha	GlcNAc	GlcA	GalA	Glc	Gal	Xyl	Fuc
PTP-1	5.48	0.30	-	0.73	-	3.00	71.77	9.03	9.70	-
PTP-2	8.27	0.51	-	1.95	0.45	9.45	50.87	12.67	15.82	-

### 3.4 Analysis of ultraviolet spectrum

**FIGURE 2:**
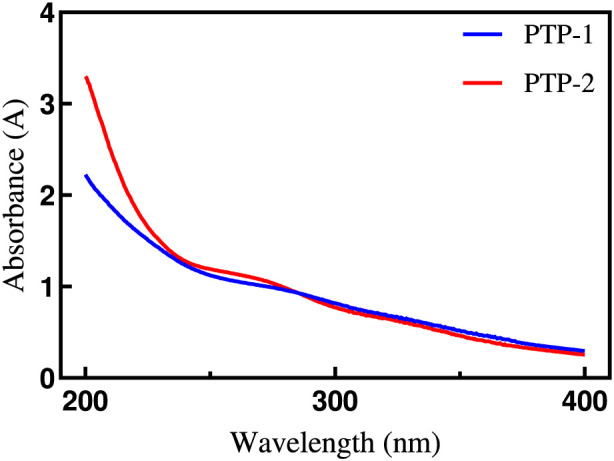
Full-spectrum UV-Vis scans of PTP-1 and PTP-2.

As shown in Figure 2, UV spectral scans of the purified polysaccharide fractions PTP-1 and PTP-2 show no significant absorbance at 260 nm or 280 nm. The absence of a detectable absorption band at 260 nm confirms minimal contamination by nucleic acids, while the lack of absorption at 280 nm indicates the absence of residual protein impurities. These results confirm the effectiveness of the purification protocol in eliminating non-polysaccharide biomolecules, such as nucleotides and polypeptides, which typically exhibit strong UV signals at these wavelengths.

### 3.5 Analysis of FT-IR spectroscopy

**FIGURE 3:**
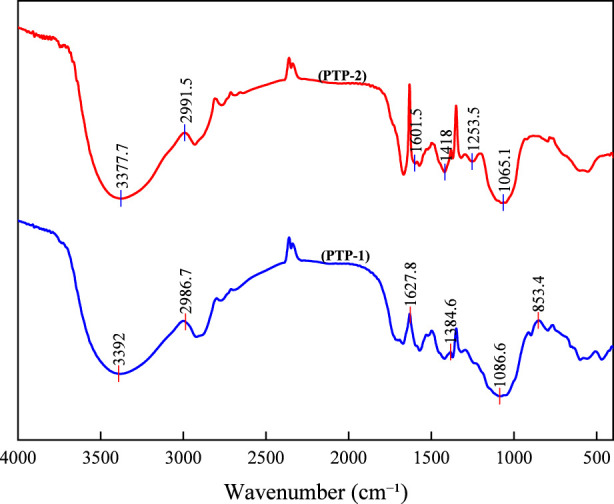
FTIR spectra of PTP-1 and PTP-2.

The FT-IR spectra of PTP-1 and PTP-2 reveal characteristic vibrational modes consistent with polysaccharide structures (Figure 3). The broad absorption band spanning 3377–3392 cm^-1^ is attributed to O–H stretching vibrations of hydroxyl groups in pyranose rings and hydrogen-bonded water molecules, in agreement with the typical infrared signatures of hydrated polysaccharides. For C–H stretching vibrations (2986–2992 cm^-1^), the asymmetric modes of aliphatic–CH_2_/–CH_3_ groups are observed, with variations in intensity reflecting distinct hydrophobic interactions between the two polysaccharides. A prominent spectral feature in the 1601–1627 cm^-1^ region corresponds to asymmetric stretching of carboxylate (-COO^-^) groups, indicative of uronic acid residues. This assignment aligns with monosaccharide compositional data, confirming the partial presence of oxidized sugar units. The fingerprint region (1045–1086 cm^-1^) displays C–O–C glycosidic bond stretching and C–O–H deformation modes, characteristic of pyranose ring systems (Hong et al., 2021). Additionally, weaker absorptions near 1384–1419 cm^-1^ may arise from O–H bending modes of adsorbed water or symmetric stretching vibrations of carboxylate anions.

The distinct spectral differences between PTP-1 and PTP-2 reflect their inherent structural divergence. Notably, the O–H stretching vibration band of PTP-2 (3377.7 cm^-1^) exhibits a redshift of approximately 14 cm^-1^ compared to PTP-1 (3392 cm^-1^), indicating strengthened intra- or intermolecular hydrogen bonding in PTP-2 (Serrafi et al., 2025). This enhancement is likely attributable to either a higher carboxyl group density (e.g., due to increased uronic acid residues) or more compact molecular packing. In the carboxyl group region (1600–1630 cm^-1^), PTP-1 displays a sharp, intense peak at 1627.8 cm^-1^, characteristic of protonated carboxylic acid (–COOH) or partially ionized carboxyl groups. In contrast, PTP-2 shows a weaker and broader absorption at 1601.5 cm^-1^, consistent with fully deprotonated carboxylate (–COO^-^) species stabilized through ionic interactions such as metal chelation or electrostatic associations (Kozioł et al., 2022). Critical disparities are also observed in the glycosidic bond region: the sharp peak at 1086.6 cm^-1^ in PTP-1 aligns with linear β-(1→4)-glycosidic linkages, while the broadened absorption near 1065 cm^-1^ in PTP-2 suggests structural heterogeneity, potential branching, or mixed α/β-configurations (Hong et al., 2021).

### 3.6 Analysis of thermogravimetric

**FIGURE 4:**
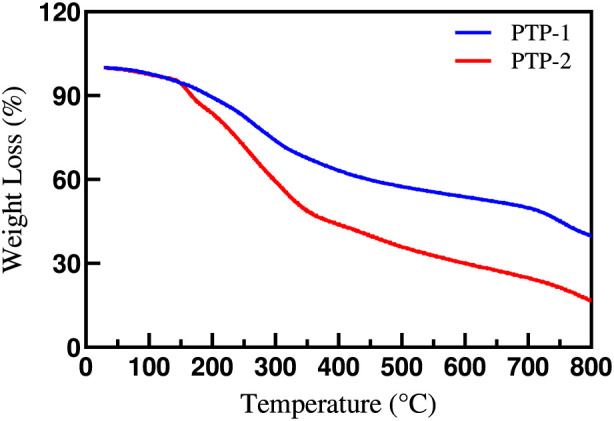
Thermogravimetric (TGA) curves of PTP-1 and PTP-2.

According to the Figure 4, thermogravimetric analysisrevealed significant differences in the thermal degradation behavior of PTP-1 and PTP-2, which are closely linked to their structural compositions. Both polysaccharides exhibited a mass loss of approximately 5%–8% within the 50 °C–150 °C range, corresponding to the evaporation of adsorbed and crystalline water. The slightly broader endothermic peak observed in PTP-1 compared to PTP-2 suggested differences in hydrophilicity or the distribution of water-binding sites, consistent with earlier FT-IR analyses that indicated lower uronic acid content in PTP-1. In the primary degradation stage, between 230 °C and 300 °C, the mass loss rate increased significantly, with cumulative losses reaching 60%–70%. PTP-2 showed a lower degradation onset temperature and a faster degradation rate compared to PTP-1, indicating that its higher uronic acid content contributed to reduced thermal stability. This difference was attributed to the decarboxylation of carboxyl groups in uronic acids, which intensified the cleavage of glycosidic bonds, while the greater abundance of acidic groups in PTP-2 further enhanced oxidative free radical formation, thereby accelerating the depolymerization of the main chain. At temperatures above 350 °C, the residual mass stabilized within the 15%–20% range, with PTP-2 producing marginally less char residue than PTP-1 during carbonization, likely due to its highly oxidized structure being more prone to complete decomposition. Together, the thermogravimetric data confirm the substantial impact of uronic acid content on the thermal stability of polysaccharides and reinforce the structural differences between the two polysaccharide components.

### 3.7 Analysis of AFM

**FIGURE 5:**
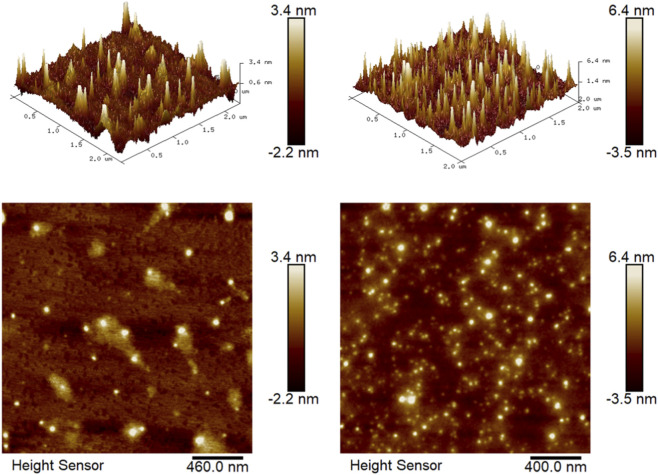
AFM topography of PTP-1 and PTP-2 (2D and 3D).

As shown in Figure 5, PTP-1 exhibits a loosely organized chain-like network in its 2D morphological profile, characterized by dispersed chains with larger interchain pores, suggesting weak intermolecular interactions. The polysaccharide surface appears smooth, with uniform brightness in the 3D height map (average height <2 nm), indicating minimal molecular stacking. These features are consistent with a predominantly linear configuration, sparse short branches, and limited hydrogen bonding. In contrast, PTP-2 displays densely packed aggregates in its 2D planar image, with reduced interchain spacing and coarse granular clusters (frequent >5 nm height domains). This structural compaction likely results from sonication-induced chain fragmentation and reorganization, which may expose hydrophobic domains to drive intermolecular clustering. The rough surface topography of PTP-2, marked by scale-like protrusions in 3D visualization, is hypothesized to arise from electrostatic shielding-mediated crosslinking of uronic acid residues (e.g., carboxyl groups in PTP-2) under sonication. Consequently, we propose distinct structural models: (1) PTP-1 adopts a linear architecture dominated by elongated backbones with infrequent short branching, while (2) PTP-2 possesses a hyperbranched topology formed by frequent interchain crosslinking of truncated branches, generating dense molecular nodes (Zhao et al., 2019).

### 3.8 Analysis of SEM

**FIGURE 6:**
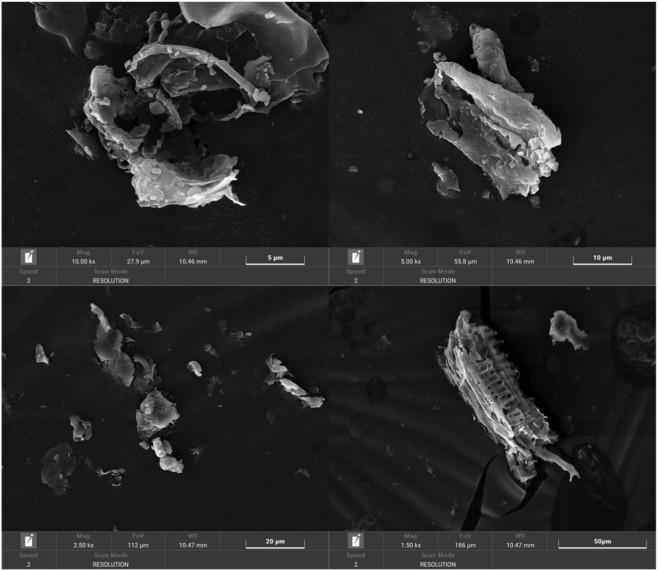
Surface morphology of PTP-1 revealed by SEM.

**FIGURE 7:**
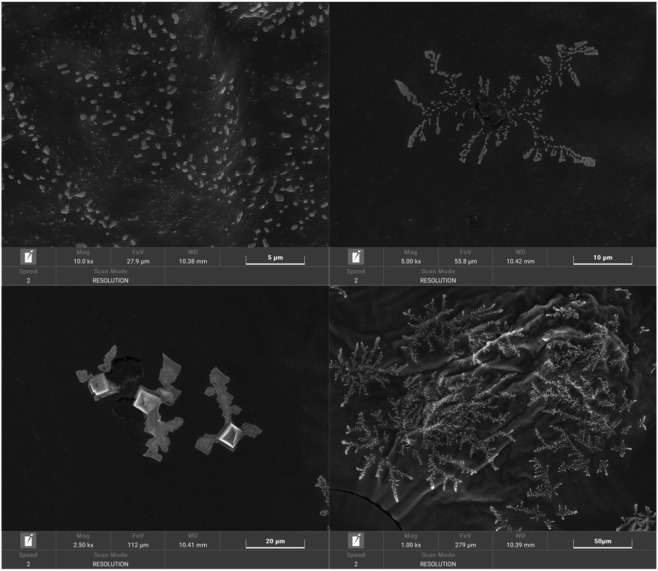
Surface morphology of PTP-2 revealed by SEM.

Scanning electron microscopy (SEM) was used to investigate the microstructural and morphological features of the polysaccharides. Figures 6, 7 present SEM images of two distinct polysaccharide types, captured at four different magnification levels. Both polysaccharides exhibit irregular, block-like morphologies, but they display distinct structural and surface characteristics. PTP-2, for example, shows dispersed dendritic structures with irregular particulate adhesion. This could be attributed to the crystalline self-assembly of sugar chains during low-temperature lyophilization, which likely influences its overall morphology. At lower magnification (scale bars of 50 μm), these particles appear heterogeneously distributed across the surface, revealing crystalline organization. Higher-resolution images (20 μm and 5 μm scale bars) expose smooth-surfaced particles with prismatic or needle-like geometries, suggesting molecular stacking patterns in solution. In contrast, PTP-1 manifests a loose skeletal framework with abundant pore networks and aggregated clusters at 50 μm magnification. Closer examination (20 μm and 5 μm scale bars) reveals rough-surfaced lamellar structures bearing granular crystalline deposits. The porous architecture of PTP-1 may enhance its water-holding capacity and adsorption properties for small molecules, whereas PTP-2’s dense crystalline features may influence dissolution behavior. These morphological discrepancies correlate with the previously observed molecular weight distribution variations between the two polysaccharides.

### 3.9 Analysis of in vitro hypoglycemic activity results

#### 3.9.1 Analysis of PTP inhibition on α-glucosidase

**FIGURE 8:**
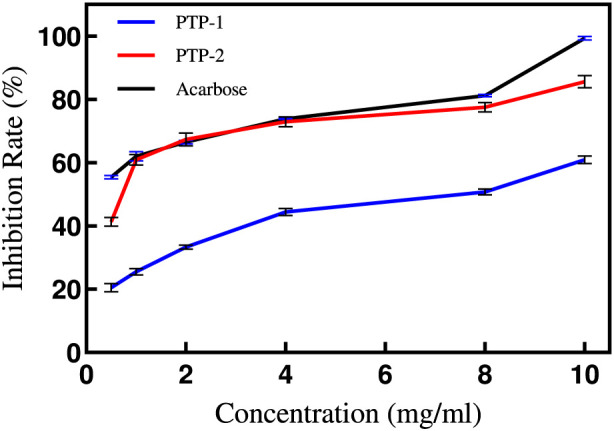
Concentration-Dependent α-Glucosidase Inhibition by PTP-1 and PTP-2.

As illustrated in Figure 8, the inhibitory effects of PTP solutions on α-glucosidase activity intensified with increasing concentrations. Notably, PTP-2 exhibited significantly stronger inhibition compared to PTP-1 (IC50 values: 6.011 vs. 0.6788). Within the 2–4 mg/mL concentration range, PTP-2 demonstrated slightly superior inhibitory capacity relative to the positive control group, whereas PTP-1 remained markedly less effective than the positive control. These findings suggest that PTP polysaccharides may regulate blood glucose levels through α-glucosidase inhibition, aligning with prior experimental observations (Ganesan and Xu, 2019). The enhanced inhibitory activity of PTP-2 versus PTP-1 likely correlates with its higher uronic acid content, as literature indicates that polysaccharides enriched in uronic acids frequently display heightened α-glucosidase inhibitory effects (Zhu et al., 2021).

#### 3.9.2 Analysis of PTP inhibition on α-amylase

**FIGURE 9:**
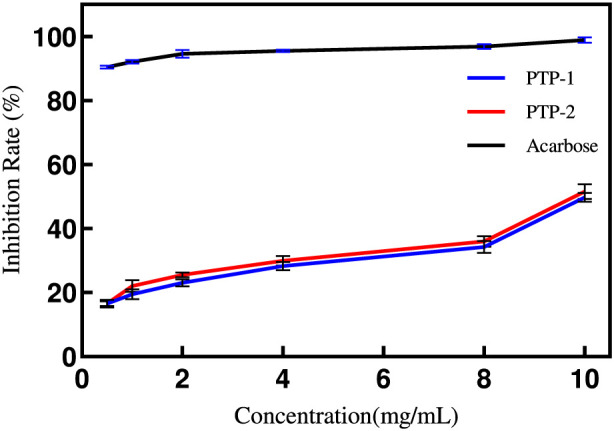
Comparative α-Amylase Inhibition: PTP-1 versus PTP-2.

Figure 9 illustrates the inhibitory effects of the two polysaccharide components on α-amylase activity. As depicted, α-amylase inhibition intensified progressively with increasing PTP solution concentrations, though the overall inhibitory effect remained below 50%. Notably, this inhibition was significantly weaker compared to the inhibition observed in the positive control group.

#### 3.9.3 The impact of PTP polysaccharides on the proliferation of human hepatocellular carcinoma cells

**FIGURE 10:**
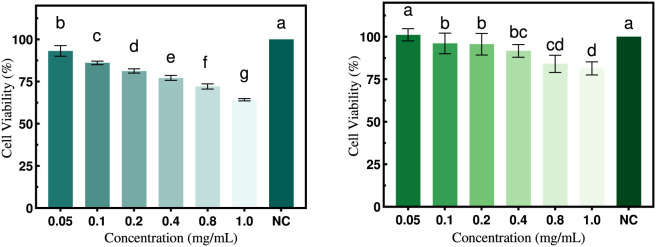
Cell viability of HepG2 cells treated with PTP-1 and PTP-2.

The cytotoxic effects of PTP components were evaluated using the CCK-8 assay to determine the optimal polysaccharide concentration. As shown in Figure 10, PTP-1 and PTP-2 exhibited cytotoxic effects on HepG2 cells at varying concentrations. Within the 50–800 μg/mL concentration range, cell viability remained above 70% after a 24-h treatment with both PTP-1 and PTP-2, indicating minimal cytotoxic effects on these cells. Hence, the concentration range of 50–800 μg/mL and a 24-h incubation period were selected for subsequent studies investigating the role of polysaccharides in ameliorating cellular insulin resistance.

#### 3.9.4 The impact of PTP on glucose uptake in insulin-resistant HepG2 cells

**FIGURE 11:**
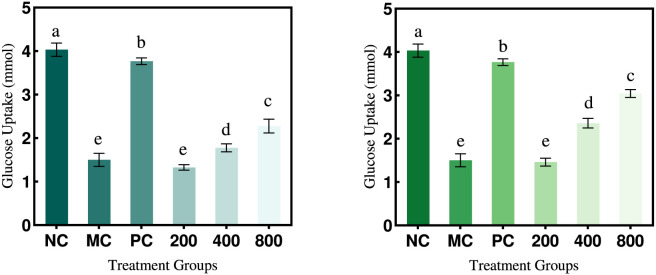
Effect of PTP-1 and PTP-2 on glucose uptake in HepG2 cells.

As shown in Figure 11, the glucose uptake in the model group decreased by 62.80% compared to the normal group, confirming the successful establishment of the insulin-resistant HepG2 cell model. Following treatment with varying concentrations of PTP-1 and PTP-2 (200-, 400-, 800-μg/mL), the glucose uptake in the treated groups increased relative to the model group. However, this increase was markedly weaker than that observed in the positive control group, with PTP-2 demonstrating superior efficacy over PTP-1 (54% increase at 800 μg/mL compared with the model group). The effectiveness of both compounds escalated in a dose-dependent manner. These results suggest that individual PTP components ameliorate insulin resistance in hepatocellular carcinoma cells. Furthermore, significant differences in efficacy between components were observed, likely attributable to variations in their monosaccharide composition and glycosidic linkage patterns.

#### 3.9.5 The impact of PTP on hepatic glycogen synthesis in insulin-resistant HepG2 cells

**FIGURE 12:**
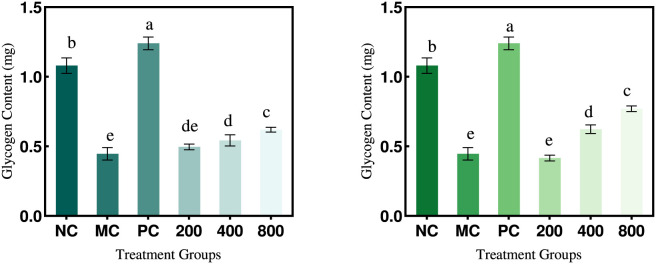
Effect of PTP-1 and PTP-2 on hepatic glycogen synthesis in HepG2 cells.

As shown in Figure 12, hepatic glycogen synthesis in the model group decreased by 53.79% compared to the normal group, reflecting impaired glycogen synthesis under insulin resistance conditions. Analysis of treatment groups demonstrated that neither PTP-1 nor PTP-2 significantly enhanced hepatic glycogen synthesis at 200 μg/mL, with minimal changes in glycogen content. When PTP-1 concentrations were increased to 400 μg/mL and 800 μg/mL, cellular glycogen synthesis showed modest increases of 8.6% and 24%, respectively, relative to the model group. In contrast, PTP-2 treatment at equivalent doses markedly elevated glycogen synthesis by 24.66% (400 μg/mL) and 54% (800 μg/mL) compared to the model group. However, these effects remained substantially weaker than those observed in the positive control group. These results align with previous findings and indicate that PTP-1 and PTP-2 can partially restore glycogen synthesis in insulin-resistant hepatocytes in a dose-dependent manner, with PTP-2 exhibiting markedly superior efficacy. The differential effects between components may arise from structural variations in monosaccharide composition and glycosidic bonds. Collectively, these data suggest that PTP components hold potential for further exploration in diabetes prevention and therapeutic strategies (Gu et al., 2022).

#### 3.9.6 The impact of PTP on reactive oxygen species levels in insulin-resistant HepG2 cells

**FIGURE 13:**
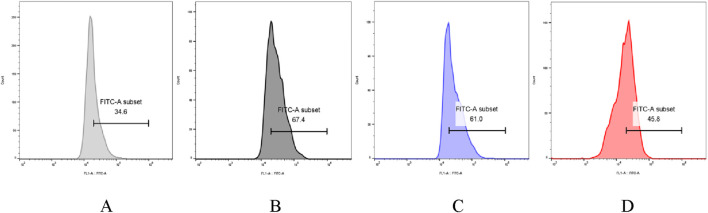
The reactive oxygen species (ROS) levels in the normal group **(A)**, the model group **(B)**, the model group with PTP-1 **(C)** and the model group with PTP-2 **(D)**.

Figure 13 demonstrates that intracellular reactive oxygen species (ROS) levels in the model group increased by 194.8% compared to the normal group, indicating a marked elevation of ROS under insulin-resistant conditions. Upon treatment of model cells with varying concentrations of PTP-1 and PTP-2, a concentration-dependent reduction in intracellular ROS levels was observed. At 800 μg/mL, ROS levels decreased by 9.5% in the PTP-1-treated group and 32.05% in the PTP-2-treated group relative to the model group. Notably, the ROS-scavenging efficiency of PTP-2 was significantly greater than that of PTP-1.

## 4 Discussion

Plant polysaccharides are important bioactive compounds found in plants, exhibiting a broad range of biological activities, including antioxidant, anti-inflammatory, immunomodulatory, anti-tumor, and hypoglycemic effects (Liu et al., 2020; Liu et al., 2025). There has been growing interest in the use of plant polysaccharides as natural therapeutic agents, especially in the pharmaceutical field, where their potential applications are gaining increasing recognition. The bioactivity of plant polysaccharides primarily stems from their unique chemical structures and molecular characteristics, which enable them to exert significant therapeutic effects in regulating the human immune system, improving metabolic functions, and addressing various pathological conditions. Compared to conventional drugs, plant polysaccharides generally have fewer side effects and a higher safety profile (Yarley et al., 2021). They are especially beneficial as therapeutic options for chronic diseases, immune disorders, and as adjunct therapies in cancer treatment. Furthermore, plant polysaccharides align more harmoniously with the body’s physiological state, reducing the risk of resistance development and minimizing toxic reactions often associated with long-term drug use. Therefore, plant polysaccharides not only complement traditional pharmaceutical treatments but also offer novel avenues for innovation and development in modern pharmacology.

Plant polysaccharides possess a range of biological activities, including antioxidant, anti-inflammatory, and immune-modulating effects. Moreover, they have been shown to reduce blood glucose levels by enhancing insulin sensitivity and promoting the repair and functional recovery of pancreatic β-cells (Deora and Venkatraman, 2022; Dou et al., 2021). In contrast to traditional pharmaceutical treatments, plant polysaccharides offer the advantage of being naturally derived, with fewer side effects, and they act through multiple physiological pathways, presenting a promising alternative for diabetes management. Despite their potential, the specific mechanisms of action of plant polysaccharides in the treatment of diabetes require further clinical investigation and scientific validation. Additionally, there are several methods for extracting plant polysaccharides, which highlights the need for standardized protocols to optimize their therapeutic effectiveness.

The polysaccharides extracted from PT were analyzed for purity, structure, composition, and appearance using techniques such as infrared spectroscopy, ultraviolet spectroscopy, high-performance liquid chromatography (HPLC), high-performance gel permeation chromatography (HPGPC), scanning electron microscopy (SEM), and atomic force microscopy (AFM). These analyses provided an initial insight into the polysaccharides derived from reed roots. The monosaccharide composition analysis revealed that the primary components were glucose, xylose, and galactose. These findings differ from those in previous studies; for instance, Zhou R utilized ultrasonic techniques to extract polysaccharides from reed rhizomes, finding that the primary monosaccharides present were galactose, fucose, and rhamnose (Zhou et al., 2020). Additionally, The molecular weights of PTP-1 (55.8 kDa) and PTP-2 (59.6 kDa) differ from those reported by Zhou et al. (2020) for reed rhizome polysaccharides (approximately 120 kDa) (Zhou et al., 2020). This suggests that different extraction methods significantly influence the types of polysaccharides obtained. Experimental validation confirmed that PTP exhibits a synergistic inhibitory effect on both α-glucosidase and α-amylase activities. The interaction between PTP and a liver cancer cell insulin resistance model was investigated to preliminarily explore the mechanism by which PTP lowers blood sugar levels. It primarily acts by restoring insulin sensitivity in cells to enhance glucose uptake while also boosting intracellular liver glycogen synthesis to decrease free blood glucose levels; Furthermore, PTP also scavenges intracellular superoxide anion, which may contribute to its blood sugar-lowering effects. These results strongly suggest that PTP holds potential as a blood sugar-lowering agent.

Overall, this study highlights the significant potential of reed root polysaccharides as a natural resource for diabetes treatment. Future research should aim to optimize extraction methods and further investigate the structure of PTP and its specific mechanisms in reducing blood sugar levels in both cellular and animal models while also assessing its safety and stability through clinical trials for practical applications.

## 5 Conclusion

In conclusion, this study successfully isolated and characterized two polysaccharide fractions (PTP-1 and PTP-2) from Phragmites communis Trin. root. PTP-2, distinguished by its higher uronic acid content (9.45%), hyperbranched structure, and superior thermal stability, demonstrated significantly stronger *in vitro* anti-diabetic potential compared to PTP-1. Its bioactivity encompassed potent inhibition of α-glucosidase, remarkable enhancement of glucose uptake and glycogen synthesis in insulin-resistant HepG2 cells, and effective reduction of intracellular ROS levels. The compelling bioactivity of PTP-2 is closely correlated with its unique structural attributes, highlighting a clear structure-activity relationship. These findings position PTP-2 as a promising multi-target natural agent for diabetes management. Future work should focus on elucidating its precise molecular mechanisms *in vivo*, conducting preclinical safety and efficacy assessments, and exploring its potential application in functional foods or nutraceuticals.

This study has several limitations. The anti-diabetic effects were evaluated solely in vitro models, which cannot fully replicate the complexity of a living organism. The precise molecular mechanisms, particularly the signaling pathways involved in insulin sensitization and ROS scavenging, require further investigation. Additionally, the potential *in vivo* bioavailability, pharmacokinetics, and long-term safety of PTP-2 remain unknown. Future research should prioritize: (1) *In vivo* validation of the hypoglycemic effects using diabetic animal models; (2) Mechanistic studies to delineate the involvement of specific pathways such as PI3K/Akt and AMPK; (3) Advanced structural analysis, including NMR spectroscopy, to fully resolve the glycosidic linkage patterns; and (4) Formulation studies to enhance the stability and delivery of PTP-2 for potential therapeutic applications.

## Data Availability

The original contributions presented in the study are included in the article/supplementary material, further inquiries can be directed to the corresponding authors.
